# Conceptual and operational definitions of the defining characteristics of the nursing diagnosis Disturbed Sleep Pattern*

**DOI:** 10.1590/1518-8345.2582.3105

**Published:** 2018-11-29

**Authors:** Juliana Prado Biani Manzoli, Marisa Dibbern Lopes Correia, Erika Christiane Marocco Duran

**Affiliations:** 1 Secretaria Municipal de Saúde, Unidade Básica de Saúde, Paulínia, SP, Brasil.; 2 Universidade Estadual de Campinas, Faculdade de Enfermagem, Campinas, SP, Brasil.; 3 Universidade Federal de Viçosa, Departamento de Medicina e Enfermagem, Viçosa, MG, Brasil.

**Keywords:** Sleep, Acute Coronary Syndrome, Nursing Diagnosis, Nursing Process, Coronary Care Units, Validation Studies

## Abstract

**Objective:**

to present the knowledge produced about sleep and Acute Coronary Syndrome in order to assist in the elaboration of the operational and conceptual definitions of the defining characteristics of the nursing diagnosis Disturbed Sleep Pattern (00198).

**Method:**

integrative review in the following databases: COCHRANE; SCOPUS; MEDLINE (Medical Literature Analysis and Retrieval System Online) via Pubmed; LILACS (Latin American and Caribbean Health Science Literature Database); CINAHL (Cumulative Index to Nursing and Allied Health Literature) and EMBASE (The Excerpta Medical Database). At the end of the search, 2827 studies were found, 43 were selected for reading, and 10 were included in the review. The gray literature was also included.

**Results:**

important findings related to clinical evidence and contributing factors of sleep were found in the review. However, in order to build definitions of the defining characteristics, it was necessary to use gray literature, such as a Portuguese dictionary and two textbooks about sleep.

**Conclusion:**

the definitions will help nurses in their practice in the collection of information, in the determination of the nursing diagnosis studied here, and in directing care measures with respect to quantity and quality of sleep of Acute Coronary Syndrome inpatients. They will also assist in the next steps of the validation of this diagnosis to the referred population.

## Introduction

Sleep is a physiological condition and has two essential patterns: Non-Rapid Eye Movement (NREM) sleep, divided into three stages - N1, N2 and N3, being the latter the deeper stage, when heart rate and blood pressure decrease; and Rapid Eye Movement (REM) sleep, when rapid eye movements, muscular hypotonia or atonia are present. These two patterns ared interspersed during the night. On a typical night of sleep, four to six NREM-REM cycles occur^(^ ^1^ ^)^.

Good sleep health with adequate quantity and quality is recognized as an indicator of vitality, mental health, and physiological, emotional, cognitive and physical well-being that leads to a good quality of life^(^ ^2^ ^-^ ^3^ ^)^. On the other hand, recurrent lack of sleep health can contribute changes in physical health, leading to serious conditions and even death^(^ ^4^ ^-^ ^5^ ^)^.

Assessing sleep health is extremely complex, but the literature^(^ ^6^ ^)^ proposes five more appropriate and relevant dimensions for measuring and defining it: duration; efficiency/continuity; sleep time in 24 hours; alertness and drowsiness; and satisfaction/quality of sleep.

Objective instruments can be used to measure sleep, including Polysomnography (PSG), the gold standard in sleep evaluation, as well as subjective instruments as self-reports and questionnaires^(^ ^7^ ^-^ ^8^ ^)^. In nursing practice, observation and reports of patients are usually useful to subjectively evaluate the patients’ sleep patterns.

In the hospital environment, several factors can disturb sleep, such as diagnostic and therapeutic procedures, the noise and illumination of the environment, and also the nursing care routine^(^ ^9^ ^-^ ^10^ ^)^.

Inpatients may have poor quality and quantity of sleep. This is the case of patients with a diagnosis of Acute Coronary Syndrome (ACS), i.e. Acute Myocardial Infarction (AMI) with or without ST-segment elevation or Unstable Angina (UA).

The literature has demonstrated the poor quality and quantity of sleep in ACS patients. A study used PSG to demonstrate that the architecture and microarchitecture of the sleep of patients in the acute phase after ACS were negatively affected and that the quality and amount of sleep was related to their quality of life in the short and long term^(^ ^11^ ^)^. Another study used a specific questionnaire and observed poor sleep quality in 71.7% of infarcted patients^(^ ^12^ ^)^.

Still in a study that sought to identify the profile of nursing diagnoses in infarcted patients, it was evidenced that the Nursing Diagnosis (ND) of Disturbed Sleep Pattern (00198) was present in 85% of participants^(^ ^13^ ^)^.

Thus, the nursing team should be sensitized on the importance of promoting quality and quantity of sleep, its contributing factors and how to promote adequate sleep for ACS patients who are hospitalized. In this way, nursing should provide care measures that evaluate, promote and maintain adequate quantity and quality of sleep in these patients^(^ ^9^ ^-^ ^10^ ^,^ ^14^ ^)^.

To this end, nurses should use tools to support an effective, rapid, direct, low-cost, and accessible bedside assessment for all patients in the hospital environment, taking into consideration also the patient’s own opinion.

In this context, the Nursing Process (NP) emerges as an alternative to sleep assessment. The NP is divided into five stages^(^ ^15^ ^)^. The second stage is the ND and serves as basis to the others; it is used by nurses to evaluate human responses after clinical judgment, with the focus on specific problems, in a state of health promotion or potential risk. There are ND taxonomies that assist in the classification and categorization of areas that concern Nursing, such as the NANDA-International Taxonomy (NANDA-I)^(^ ^15^ ^)^.

In order to determine a ND, nurses should know their indicators, which include the Defining Characteristics (DC) and their contributing factors, corresponding to the Related Factors (RF)^(^ ^15^ ^)^.

The DC are clinical manifestations, evidences, signs and symptoms or inferences that can be observed in the individuals to determine a given ND. However, in clinical practice, nurses may face lack of clarity regarding what and how to evaluate.

Thus, validation studies of ND have been proposed in order to contribute to lessen the variability present in clinical situations and to help in the accurate identification of ND that corresponds to the clinical condition presented by the patient^(^ ^16^ ^-^ ^17^ ^)^.

In order to carry out the validation of a ND, a first step is proposed, namely, the construction of conceptual definitions (CD) and operational definitions (OD) of the DC of a given ND for a specific population^(^ ^16^ ^)^. CD correspond to the theoretical meaning of the DC. On the other hand, the OD should assign a meaning capable of communicating how a given concept is applied, that is, they should elucidate the practical meaning of the CD of each DC and specify what practical activities or procedures are necessary for the evaluation of such characteristics^(^ ^18^ ^-^ ^19^ ^)^.

Therefore, the present study proposed to carry out an Integrative Review with the objective of presenting the knowledge produced about sleep and acute coronary syndrome in order to assist in the elaboration of the operational and conceptual definitions of the defining characteristics of the Nursig Diagnosis of Disturbed Sleep Pattern (00198).

## Method

Integrative Review (IR) is a method that aids the construction of the CD and OD of the DC of a ND. It allows obtaining sources of knowledge about a particular problem and must follow rigorous methodological standards. An IR provides the reader with subsidies for the practice and advancement of nursing. Six phases must be followed in an IR, namely: identification of the theme or question; sampling or search in the literature; categorization of studies; evaluation of the included studies; interpretation of results; synthesis of the knowledge found in the articles analyzed or presentation of the IR^(^ ^20^ ^)^.

The present IR was guided by the questions “What are the clinical evidences of ACS patients who present altered sleep patterns?” and “What are the contributing factors for altering the sleep pattern in ACS patients?”.

The IR was carried out between December 22 and 27, 2016. The search of literature was made in the following databases: COCHRANE, SCOPUS, Medical Literature Analysis and Retrieval System Online (MEDLINE via Pubmed) with Medical Subject Headings of United States National Library of Medicine (MeSH), Latin American and Caribean Health Science Literature Database (LILACS) with Health Sciences Descriptors (DeCS), The Cumulative Index to Nursing and Allied Health Literature (CINAHL) with CINAHL titles and The Excerpt Medical Database (EMBASE) with Emtree.

The descriptors used in the various databases were: “disorders of sleep onset and maintenance”; “sleep deprivation”; “sleep disorders by excessive sleepiness”; “circadian rhythm sleep disorders”; “sleep-wake disorders”; “intrinsic sleep disorders”; “dyssomnias”; “sleep-wake transition disorders”; “sleep waking disorders”; “REM sleep behavior disorder”; “sleep apnea syndromes”; “sleep pattern disorders”; “coronary disease”; “acute coronary syndrome”; “coronary care units”; “unstable angina” and “myocardial infarction”. The Boolean operators “OR” and “AND” were used to cross the descriptors and keywords.

The inclusion criteria were articles coducted with patients aged 18 years or older and diagnosed with ACS regardless of whether they had undergone surgical or conservative intervention. The IR considered studies addressing clinical indicators and contributing factors of disturbed sleep pattern in the patients in question, hospitalized in Cardiac Inpatient Units or Coronary Units, published in English, Portuguese or Spanish, with no delimitation of date of publication.

The exclusion criteria were articles in editorial formats, letters to the reader, and abstracts of congresses. Articles that addressed the relationship between obstructive sleep apnea and coronary artery disease as the cause of ACS or those that did not include ACS patients were also regarded as exclusion criteria. Such choice was made by the authors to maintain the focus of the search only on the findings of clinical evidence and contributing factors of sleep disorders in ACS, and not on the factors that contributed to the occurrence of ACS.

An instrument created and validated in Brazil was used to extract data from the selected articles, including identification data, study institution, journal characteristics, study methodology and methodological rigor^(^ ^21^ ^)^.

After reading and extracting the data, two tables with information of the articles were created. The information was the title, journal of publication, country of origin of the study, language and year of publication, methodological delineation, level of evidence and objectives.

The level of evidence was classified in seven levels, namely, level I - evidence from systematic review or meta-analysis covering all relevant randomized controlled trials, or from systematic reviews whose clinical trials had been subjected to randomization and control; level II - evidence from at least one well delimited randomized controlled trial; level III - evidence from a designed and controlled, but not randomized study; level IV - evidence from cohort studies or case-control studies; level V - evidence from systematic review of descriptive and qualitative studies; level VI - evidence from a descriptive or qualitative study; level VII - evidence from authoritative opinion or expert reports^(^ ^22^ ^)^.

A total of 2827 results were found in the search. Articles were pre-selected by evaluation of the titles and, when necessary, the abstracts. Sixty-one articles were kept after pre-selection. Of these, 18 were duplicated in other databases and were therefore excluded. There were still 43 articles, which were reevaluated after a quick reading to ensure compliance with the established criteria. Articles that did not meet the inclusion criteria were excluded. Other reasons for non-selection were lack of specification in the study whether the patients had ACS, and lack of relevant content of clinical evidence and/or contributing factors to Disturbed Sleep Pattern (00198) in these patients. Due to these reasons, 33 articles were excluded, leaving a final sample of 10 articles.

**Figure 1:**
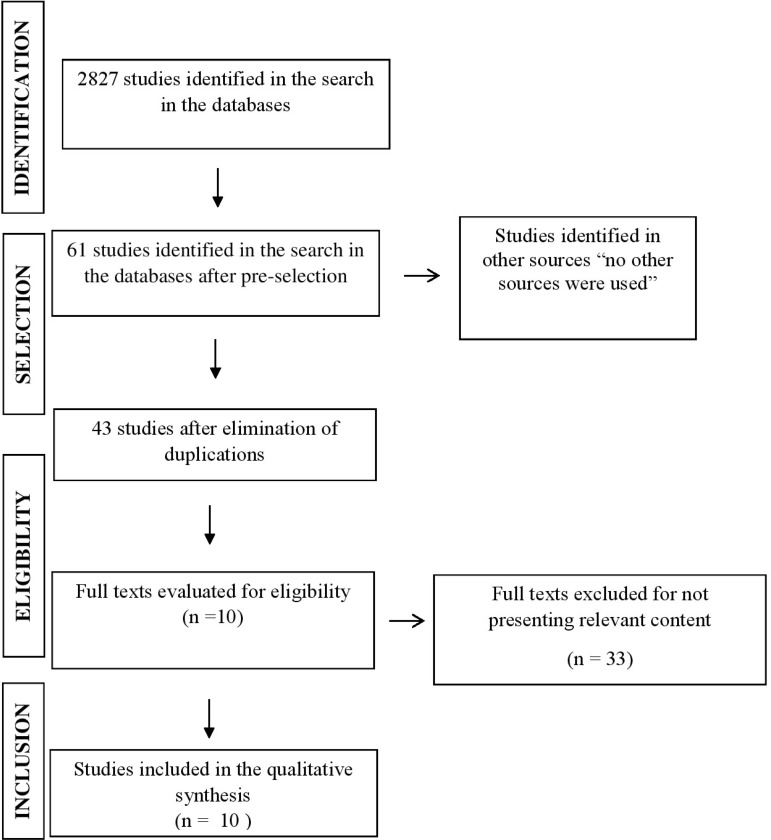
– Flowchart used in the selection of studies in this Integrative Review. Campinas, SP, Brazil, 2018

Figure 1 presents the flowchart of article selection, adapted from the Preferred Reporting Items for Systematic Reviews and Meta-Analyses – PRISMA 2009^(^ ^23^ ^)^.

At the end of IR, the gray literature was also explored, since it was not possible to construct the conceptual and operational definitions with the articles of the IR.

## Results

**Figure 2:**
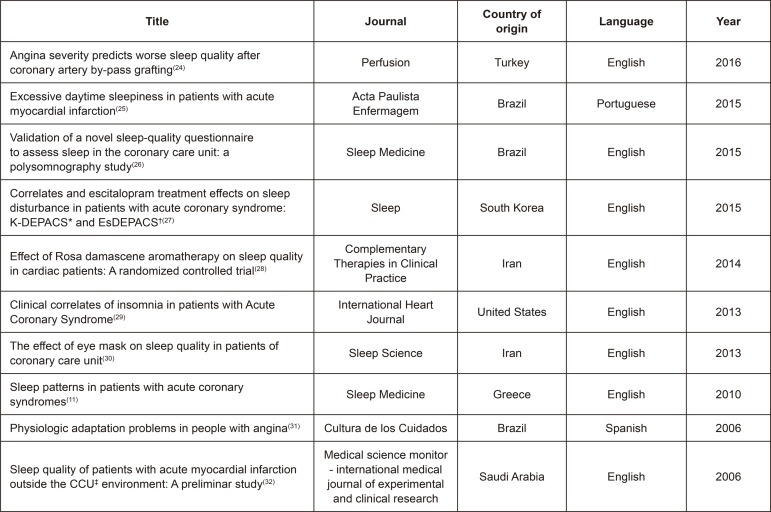
– Presentation of articles selected according to title, journal of publication, country of origin of the study, language and year of publication. Campinas, SP, Brazil, 2018

**Figure 3:**
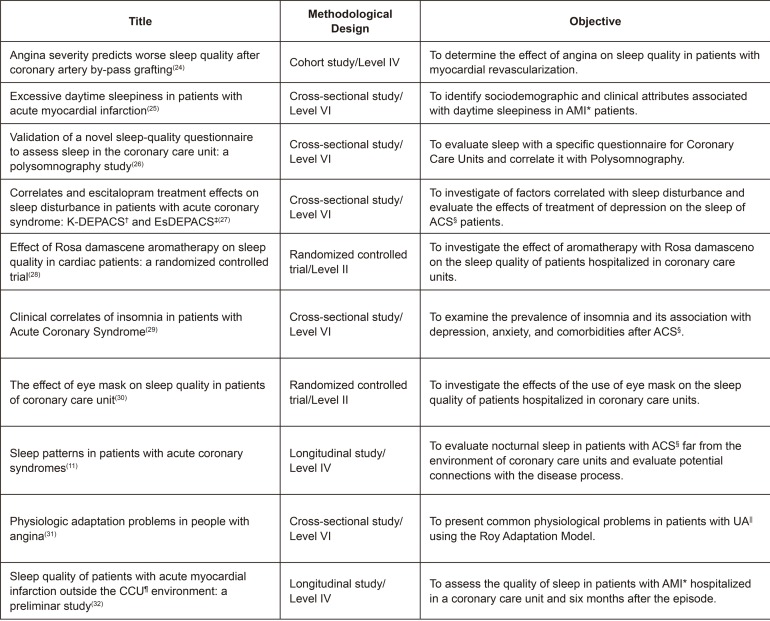
– Presentation of articles according to study title, methodological design, and objective. Campinas, SP, Brazil, 2018

The final sample consisted of ten studies. The main characteristics of the articles are described in Figures 2 and 3.

*K-DEPACS- Korean DEPression in Acute Coronary Syndrome; †EsDEPACS- Escitalopram for DEPression in Acute Coronary Syndrome; ‡CCU- Coronary Care Units

*AMI - Acute Myocardial Infarction; †K-DEPACS - Korean DEPression in Acute Coronary Syndrome; ‡EsDEPACS - Escitalopram for DEPression in Acute Coronary Syndrome; §ACS- Acute Coronary Syndrome; ||UA - Unstable Angina; ¶CCU - Coronary Care Units

Regarding the main results of the articles included in the IR, they identified that patients with recent AMI had worse quality and quantity of sleep^(^ ^11^ ^,^ ^24^ ^,^ ^32^ ^)^ and that a high angina score and length of stay in intensive care unit were predictors of poor sleep quality^(^ ^24^ ^)^. They also pointed out that during the hospitalization more than one third of the ACS patients had insomnia^(^ ^29^ ^)^. And patients with angina had disturbed sleep pattern due to pain^(^ ^31^ ^)^.

Another study identified that infarcted patients with Body Mass Index (BMI) over 30 kg/m^2^ and clinical worsening had a worse score on the Epworth Sleepiness Scale and that 29.2% of the patients presented daytime sleepiness^(^ ^25^ ^)^.

Studies on non-pharmacological interventions such as aromatherapy^(^ ^28^ ^)^ and use of eye masks^(^ ^30^ ^)^ and pharmacological interventions such as the use of Escitalopram^(^ ^27^ ^)^ showed positive results, in which there was a significant improvement in the sleep of the study patients.

The study that sought to evaluate sleep through a specific questionnaire and compare it to PSG findings identified a correlation with sleep efficiency, REM sleep and excitation index, but the minority of the patients were classified as poor sleepers^(^ ^26^ ^)^.

**Figure 4:**
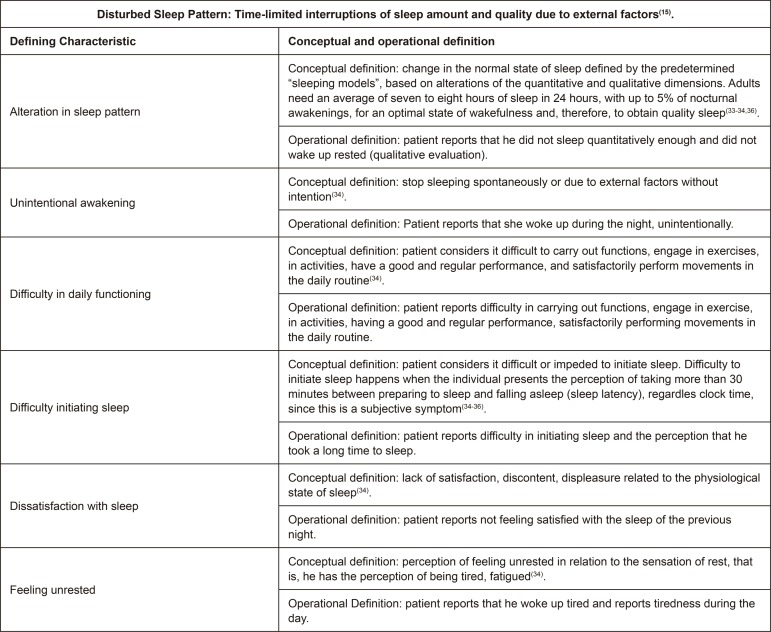
– Conceptual and operational definitions of the Defining Characteristics of the Nursing Diagnosis Disturbed Sleep Pattern. Campinas, SP, Brazil, 2018

Important findings related to sleep and ACS were evident in the present IR. However, they were not sufficient for the construction of the CD and OD of the DC of Disturbed Sleep Pattern (00198), which justified the use of gray literature. Thus, other sources were searched, including a dictionary of the Portuguese language and two textbooks on sleep^(^ ^33^ ^-^ ^35^ ^)^ and an article^(^ ^36^ ^)^ found that was not included and analyzed in the IR. The OD were developed by the authors based on the information identified in the gray literature. The Figure 4 below presents the CD and OD of the DC Disturbed Sleep Pattern (00198).

## Discussion

In this study, clinical findings were the findings related to sleep assessments through instruments such as PSG and/or validated sleep questionnaires.

The Pittsburgh Sleep Quality Index (PSQI) was used to evaluate sleep in a study with patients who underwent artery bypass surgery after myocardial infarction, recent or not. The main findings related to sleep were that the PSQI global score was higher in patients with recent myocardial infarction, presenting worse scores when evaluated in relation to sleep duration, sleep latency, sleep efficiency and overall sleep quality. High angina scores were still significantly correlated with poor sleep duration, sleep disturbances, and total score of the questionnaire. As the main predictors of poor sleep quality, high angina scores and long length of stay in Intensive Care Unit were found^(^ ^24^ ^)^.

There was a correlation of poor sleep quality with UA in another study, showing that sleep apnea, daytime sleepiness, tiredness, lethargy, nocturnal awakenings, difficulty sleeping and superficial sleep were associated with the use of sedative drugs, tension due to illness, fear and anxiety^(^ ^31^ ^)^.

Pain was also identified as a factor that disturbs sleep in a study with hospitalized patients. Other factors such as excessive lighting, nursing care, and organic disorders such as fatigue were also highlighted^(^ ^9^ ^)^.

In an evaluation of the sleep of patients in Coronary Units, patients were submitted to PSG in a place where the environmental factors were controlled, and six months after the event, on an outpatient basis. Despite the controlled environmental factors in the hospital, patients presented significantly increased spontaneous excitation, waking hours, latency, and decreased efficiency in REM sleep when compared to the six months of after AMI control, demonstrating that poor sleep quality was strongly related to the disease and not only to environmental factors^(^ ^32^ ^)^.

A similar result was presented in a study that assessed sleep quality in ACS patients in three days, one month and six months after the event, outside the Coronary Care Unit environment to make it possible to evaluate the connections of sleep dysfunction with the disease process by PSG. The study showed that, over time, there was a significant improvement in PSG parameters, with an increase in the total sleep time, efficiency, slow wave sleep, and decreased excitation and wakefulness after sleep onset. Six months after the event, the sleep architecture was normal, suggesting that the major cause of sleep disorders was the underlying acute disease^(^ ^11^ ^)^.

On the other hand, a study that examined the prevalence of insomnia in ACS patients, hospitalized and after hospitalization, and its association with depression, anxiety and comorbidities found that 37% of the patients who reported moderate or severe insomnia during hospitalization presented 76 minutes more of wakefulness after the onset of sleep at home. For this evaluation, the patients were submitted to questionnaires to assess insomnia, sleepiness, depression and anxiety at admission, and weeks later, outpatient PSG. Other findings were that patients with insomnia presented higher scores in depression and anxiety questionnaires (p < 0.01) when compared to non-insomniacs, but no evidence was related to the sleepiness scale, and the scores on the sleepiness scale were positively correlated with depression scores (p = 0.024)^(^ ^29^ ^)^.

Still on the relationship bewteen sleep disorders and depressive symptoms, a research investigated sleep disorders in ACS patients and the effect of treatment of depression with Escitalopram on their sleep. Sleep disturbance was assessed by the Leeds Sleep Evaluation Questionnaire which evaluates “initiation of sleep”; “sleep quality”, “awakening from sleep”, and “behavior after awakening”. The main factors associated with the total score of the sleep disorder questionnaire were depressive symptoms, older age, female gender, hypertension and severe ACS^(^ ^27^ ^)^.

Two studies, which follow, have used non-drug interventions in patients with ACS to improve sleep quality. They were included in the IR because they evaluated sleep disorders and their possible contributing factors.

One of them used the PSQI to evaluate the sleep of inpatients, most of them diagnosed with ACS, after a intervention with a specific flavouring preparation. Patients in the control and experimental groups presented alterations related to latency, duration, and habitual efficiency of sleep and to the domains of sleep disorders, which decreased significantly after the intervention (p < 0.05)^(^ ^28^ ^)^.

Other study evaluated the effect of the use of eye mask on sleep quality of patients who had Myocardial Infarction, chest pain and angina and were hospitalized in Coronary Care Units, using the Verran Snyder-Halpern Sleep Scale. The study showed that patients had sleep disorders related to sleep disturbance, efficiency and supplementation. The use of masks caused an improvement in the subscale disturbance, with changes related to the item “wake up after the onset of sleep”; in the subscale efficiency, related to sleep quality and sleep adequacy assessment; and in the subscale supplementation, where the changes were related to “waking up after final excitement”^(^ ^30^ ^)^.

These findings demonstrate that sleep disorders are related to both underlying disease and environmental factors. In line with this, a study carried out in an Intensive Care Unit demonstrated that the causes of sleep deprivation are related to both intrinsic factors - acute condition of the disease - and environmental factors. Interventions to minimize sleep disorders may promote improved sleep quality of inpatients^(^ ^37^ ^)^.

A study created a tool to evaluate the sleep of ACS patients hospitalized in coronary units. This tool was compared to PSG. The main finding was related to the contributing factors of poor sleep quality, namely exposure to light and concern with the disease in the nighttime, conversation of hospitals workers, and poor bed quality during daytime sleep. Overall results showed that patients admitted to Coronary Care Units were classified as “poor sleepers” (22%); “regular sleepers” (43%) and “good sleepers” (35%)^(^ ^26^ ^)^.

Other clinical evidence was found in a study that characterized daytime sleepiness and identified clinical and sociodemographic characteristics in AMI patients using the Epworth Sleepiness Scale. The study identified excessive daytime sleepiness in 29.2% of the patients. As contributing factors, the age group (60 years or older presented a 3.43-fold higher chance than individuals under 60 years); marital status (separate individuals presented a 9.23-fold higher chance than those of another civil state) and BMI (individuals with BMI greater than 30 kg/m^2^ had a 5.9-fold higher chance than individuals with BMI equal to or less than 30 kg/m^2^)^(^ ^25^ ^)^.

Age is the factor that most influences the sleep-wake rhythm. Age determines the time of each sleep state. This variable must be considered whenever reference is made to the temporal dimension of sleep. There is evidence that the sleep of the elderly has distinct characteristics compared to young adults. In the elderly, the REM stage decreases, stage changes are frequent with alternations between superficial and deep sleep, and the waking times increase^(^ ^33^ ^)^.

Although the findings of this IR were important, the gray literature had to be used to achieve the objectives of the study. This was due to the fact that the articles did not present enough information for the construction of the CD and OD of the ND in question. However, all the phases proposed by the literature for the conceptualization of DC were followed, which made the study feasible.

This was seen as an obstacle to the realization of the IR as the first step in validation studies. Most of the scientific articles on original researches did not address clinical evidences and contributing factors so as to conceptualize them; it is, therefore, difficult to gather quantitative and qualitative material to review a particular ND^(^ ^38^ ^)^.

## Conclusion

After reviewing the articles that evaluated the sleep of ACS patients through questionnaires, PSG (gold standard), interviews and physical examination, the main clinical evidence related to sleep of hospitalized ACS patients in the studies corroborate with the DC and RF described in NANDA-I.

The clinical evidences found were generally short sleep duration, total sleep time decreased, nocturnal awakenings, wakefulness after onset of sleep, superficial sleep, reductions in the efficiency of REM sleep, sleep deprivation, difficulty in sleeping, reduced sleep latency, reduced sleep efficiency and overall sleep quality, daytime sleepiness, fatigue, lethargy, increased spontaneous excitation, increased wakefulness time related to intrinsic factors such as acute phase of ACS, depression and anxiety, as well as environmental factors.

Among the contributing factors were those related to the disease itself, pain, long length of stay, use of sedative drugs, stress related to the disease, fear, anxiety, environmental factors such as excessive lighting, nursing care, and organic disorders such as fatigue and depressive symptoms, old age, female gender, hypertension and severe ACS.

The present study will contribute to the accomplishment of the next steps of the process of validation of this diagnosis to ACS patients and it will also assist in the determination of the ND in question by the nurses in the clinical practice. Using the CD and OD as an instrument to identify changes in the sleep pattern, nurses can plan the care directed to this need, contributing to the improvement of the quality and quantity of sleep of ACS patients who are hospitalized.

It is recommended that further studies be conducted to establish the profile of the ND Disturbed Sleep Patter (00198) in the population of ACS patients who are hospitalized, bearing in mind that these type of studies are still scarce.

The absence of conceptual definitions of the contributing factors was an option of the researchers. Although having identified them in the IR, the authors had the objective of limiting the study in this first moment to the presentation of clinical evidences and their respective definitions in the study population. This is so because this is the first phase of a validation study of the aforementioned diagnosis for the population of ACS patients. The next phases, i.e. content analysis and clinical validation, are already in progress, but do not include such contributing factors.

Further studies addressing the determinants of this ND are therefore recommended.
